# TWEAK/Fn14 Drives Tumor Progression and Is Associated With Poor Survival of Colorectal Liver Metastases With Replacement Growth Patterns

**DOI:** 10.1002/cam4.71027

**Published:** 2025-07-09

**Authors:** Naoki Matsuyama, Takanori Konishi, Tsukasa Takayashiki, Shigetsugu Takano, Daisuke Suzuki, Nozomu Sakai, Isamu Hosokawa, Takashi Mishima, Kensuke Suzuki, Hitoe Nishino, Shinichiro Nakada, Masayuki Ohtsuka

**Affiliations:** ^1^ Department of General Surgery Chiba University Graduate School of Medicine Chiba Japan

**Keywords:** colorectal liver metastases, Fn14, histopathological growth pattern, TWEAK

## Abstract

**Objective:**

Colorectal liver metastases (CRLMs) with replacement growth pattern (rHGP) are associated with a poor prognosis; however, the underlying mechanisms driving rHGP remain poorly understood. This study investigated the effect of the TWEAK/Fn14 axis in CRLMs on tumor progression to explore the pathology of CRLMs with rHGP.

**Method:**

In total, 129 patients with CRLMs who underwent curative resection were investigated.

**Results:**

Patients with rHGP had significantly poor overall survival after surgery than those with other growth patterns. CRLMs with rHGP exhibited epithelial‐mesenchymal transition (EMT) activation and a distinct immune microenvironment. We focused on TWEAK/Fn14 axis to clarify the mechanism by which the tumor microenvironment affects tumor progression in CRLMs with rHGP. TWEAK was expressed in the tumor microenvironment, accompanied by the infiltration of Th17 cells and M2 macrophages, whereas Fn14 was expressed in cancer cells within CRLMs. High TWEAK/high Fn14 expression in CRLMs was more frequently observed in CRLMs with rHGP and was associated with a worse prognosis. This was accompanied by high grade of tumor budding and poorly differentiated clusters in CRLMs, and increased risk of extrahepatic metastases. In vitro, recombinant TWEAK (100 ng/mL) induced phosphorylation of IκB and NFκB (p65) and also enhanced cell migration, invasion, and EMT maker expression in colorectal cancer cells. Cell migration and invasion enhanced by recombinant TWEAK were suppressed by an Fn14 antagonist (ITEM‐4: 200 ng/mL).

**Discussion:**

This study clarified that the TWEAK/Fn14 axis in CRLMs promotes invasiveness and metastatic potential, leading to poor prognosis. It may be crucial in the adverse survival outcomes of CRLMs with rHGP.

## 1 Introduction

Colorectal cancer (CRC) is one of the most common malignancies worldwide [1]. CRC cells acquire migratory and metastatic ability through epithelial‐mesenchymal transition (EMT) [2, 3]. Distant metastases develop in approximately 50% of patients with CRC, and the liver is the most common metastasis site [4, 5]. Numerous studies have revealed the mechanisms underlying tumor dissemination and development of metastases from CRC [6, 7]. In contrast, the biological characteristics of colorectal liver metastases (CRLMs) are not fully understood, and the progression of CRLMs may affect patient survival. Therefore, the biological phenomena occurring within CRLMs and their effects on tumor progression must be further investigated.

Previously, we reported the presence of a microscopical fibrous pseudocapsule between liver metastases and the surrounding hepatic parenchyma as a favorable prognostic factor in patients with CRLMs [8]. This finding laid the groundwork for subsequent research on histopathological growth patterns (HGPs). An international consensus guideline proposed three common HGPs: desmoplastic growth pattern (dHGP), replacement growth pattern (rHGP), and pushing growth pattern (pHGP) [9, 10]. Previous studies have demonstrated that patients with rHGP, the most common pattern in CRLMs, have a poor prognosis after liver resection [11]; therefore, the biological mechanism underlying rHGP needs to be further elucidated to improve the prognosis of patients with CRLMs. CRLMs with rHGP obtain blood supply through pre‐existing liver sinusoidal vessels [12]. This vascularity, known as vessel co‐option, indicates that CRLMs with rHGP may have a specific tumor microenvironment mediated by infiltrating inflammatory cells through the sinusoids. Moreover, cytokines released from inflammatory cells in the tumor microenvironment of rHGP may be important in tumor progression in CRLMs.

Tumor necrosis factor (TNF)‐like weak inducer of apoptosis (TWEAK) is a cytokine commonly released from inflammatory cells, and its sole receptor fibroblast growth factor‐inducible 14 (Fn14) is expressed in damaged and cancerous cells [13, 14]. TWEAK modulates the tumor microenvironment through its interaction with Fn14, leading to the release of chemokines and the recruitment of immune cells [15]. The TWEAK/Fn14 axis is also involved in tumor progression, including cancer proliferation, migration, and metastasis [16, 17]. The effects of TWEAK and Fn14 on tumor progression in CRC are controversial. A previous study indicated that TWEAK in CRC cancer inhibits tumor migration, leading to a favorable prognosis [18]. However, another study demonstrated that the TWEAK/Fn14 axis in CRC promotes liver metastases [19]. Upregulated Fn14 RNA expression was previously detected in CRLMs with rHGP by RNA sequencing [20]; therefore, we hypothesized that the TWEAK/Fn14 axis in CRLMs contributes to tumor progression and causes the poor prognosis of CRLMs with rHGP. This study aimed to clarify the pathological characteristics of CRLMs with rHGP and examine the effect of the TWEAK/Fn14 axis in CRLMs on tumor progression and prognosis.

## 2 Materials and Methods

### 2.1 Patients

In total, 129 patients with CRLMs who underwent curative liver resection at Chiba University Hospital between 2008 and 2018 were retrospectively analyzed. All patients provided a written informed consent according to the Declaration of Helsinki. The Committee on Human Research of Chiba University School of Medicine approved the study protocol (Approval code: #M10528).

### 2.2 Histopathological Growth Patterns

**FIGURE 1:**
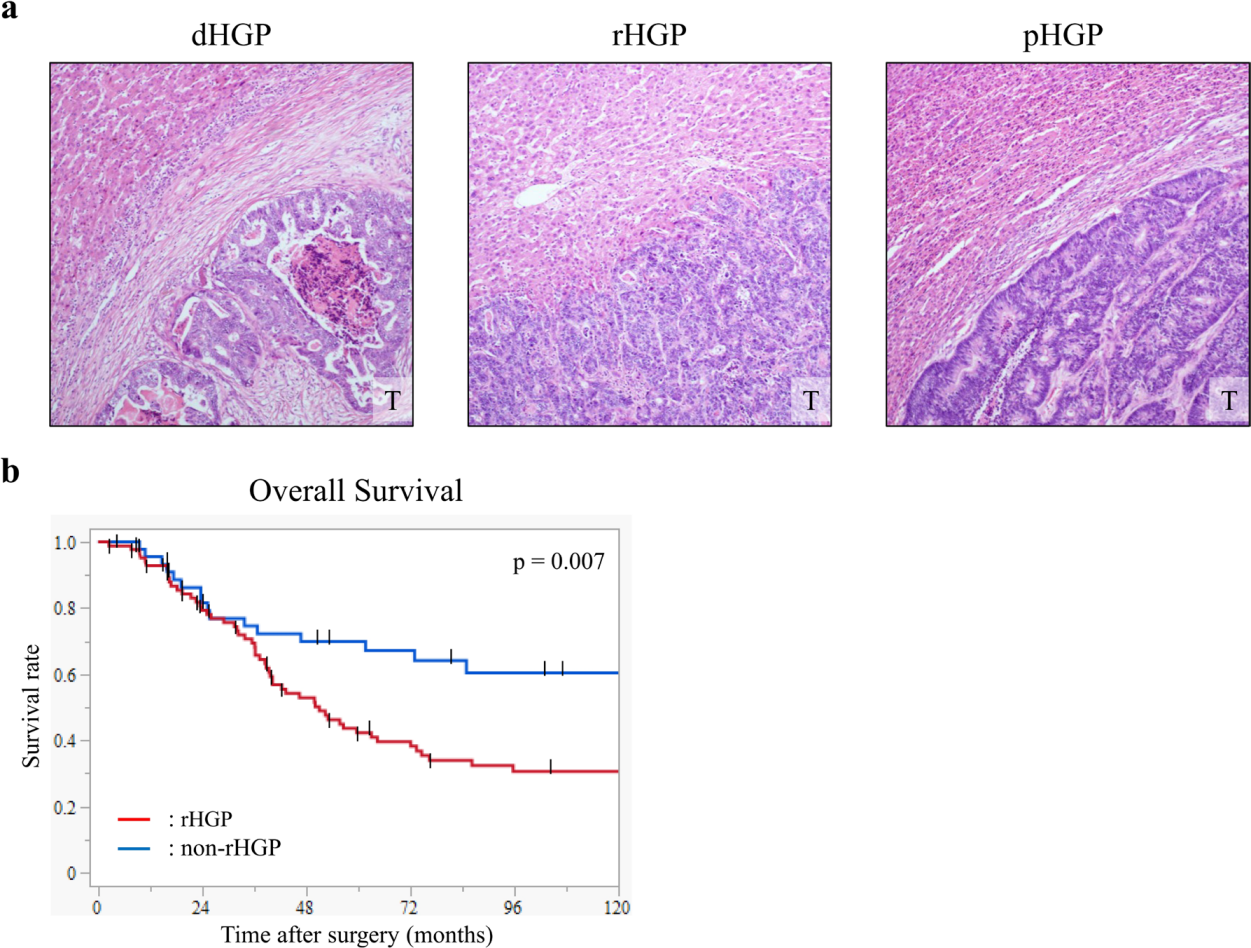
(a) Representative image of the histopathological growth patterns (HGPs) in CRLMs (T: Tumor). dHGP, desmoplastic growth pattern; rHGP, replacement growth pattern; pHGP, pushing growth pattern. (b) The overall survival rate following liver resection of the patients with rHGP (*n* = 85) was compared with that of patients with other HGPs (non‐rHGP, *n* = 44) by the Kaplan–Meier method.

CRLM tissue specimens were sliced from formalin‐fixed paraffin‐embedded (FFPE) blocks. Sections were stained with hematoxylin and eosin (H&E) for histological examination. HGPs of each case were determined according to the international consensus guideline (Figure 1a) [9, 10]. Briefly, in dHGP, the metastasis was separated from the liver tissue by a desmoplastic rim. In rHGP, cancer cells form cell plates that are in continuity with the liver cell plates. In pHGP, the liver cell plates are compressed and pushed away by the metastases. The percentage of HGPs at the interface between the liver metastases and liver parenchyma was evaluated and scored. Each case was categorized as a distinct HGP that comprises ≥ 50% of the tumor‐liver interface. If no HGPs were predominant, the case was defined as a mixed type.

### 2.3 Tumor Budding and Poorly Differentiated Clusters

The grading classification for tumor budding (TB) was conducted according to the 9th edition of the Japanese Classification of Colorectal Carcinoma [21]. All liver metastases were assessed by H&E staining and classified into four grades on the basis of the number of budding per 200× high‐power field as follows: grade0 (G0), no budding; grade1 (G1), < 5 budding; grade2 (G2), 5–9 budding; and grade3 (G3): ≥ 10 budding. Poorly differentiated clusters (PDCs) in CRLMs were evaluated on the basis of the criteria in primary tumors as previously described [22, 23]. Briefly, PDCs were defined as cancer cell clusters with poor glandular formation comprising ≥ 5 cancer cells. Liver metastases were categorized into four grades on the basis of the number of PDCs per 200× high‐power field as follows: grade0 (G0), no PDCs; grade1 (G1), < 5 PDCs; grade2 (G2): 5–9 PDCs; and grade3 (G3), ≥ 10 PDCs.

### 2.4 Immunohistochemistry

CRLM tissue specimens were sliced from FFPE blocks. Microwave antigen retrieval was performed in citric acid buffer at 100°C for 25 min. Endogenous peroxidase activity was blocked with 3% hydrogen peroxide in methanol for 15 min. Non‐specific protein binding was minimized with 5% bovine serum albumin at room temperature for 15 min. Sections were then incubated with primary antibodies shown in Table S1. Sections were washed and incubated with secondary antibodies (EnVision kits; K4001 and K4003; Dako), and then stained with a peroxidase DAB kit (Nacalai Tesque Inc.). The CRLMs with TWEAK expression at the invasive front of the tumor were defined as high TWEAK expression, whereas the CRLMs with undetectable or minimal TWEAK expression at the invasive front of the tumor were defined as low TWEAK expression. The CRLMs whose Fn14 expression was the same as or stronger than the staining intensity of the vessels, as an internal control, were defined as high Fn14 expression, and the CRLMs whose Fn14 expression was undetectable or weaker than the internal control were defined as low Fn14 expression. The expression of CD8, IL‐17, and CD163 was evaluated by counting positively stained inflammatory cells at the invasive front of the tumor across an average of five different high‐power fields (×400). The results were classified as positive or negative according to the cut‐off values determined by receiver operating characteristic analysis on the basis of their association with TWEAK positivity (CD8; 35 cells/field, IL‐17; 29 cells/field, CD163; 13 cells/field). For patients with multiple CRLMs, a representative metastasis exhibiting the assessed HGP was selected for evaluation. The immunohistochemical staining was independently evaluated by two investigators. In case of disagreement, a third investigator reviewed the staining and provided a final judgment.

### 2.5 Cell Lines and Cell Culture

Human CRC cell lines DLD‐1 (CCL‐221) and WiDr (CCL‐218) were purchased from the American Type Culture Collection and were cultured in the appropriate medium as recommended by the supplier and fetal bovine serum (FBS).

### 2.6 Proliferation Assay

CRC cell lines were seeded in 96‐well microplates at a density of 3000 cells/well. 10 μL of Cell Count Reagent SF (Nacalai Tesque Inc.) was added to each well. After incubation for 1 h at 37°C, the absorbance at 450 nm was measured.

### 2.7 Migration and Invasion Assays

Migration and invasion assays were conducted using CRC cell lines. For the migration assays, 3 × 10^5^ cells were seeded into the upper chamber of a transwell insert (8 μm pore size, Corning Falcon) in a serum‐free medium. The lower chamber contained medium supplemented with 10% FBS under the following conditions: (i) phosphate‐buffered saline control, (ii) 100 ng/mL recombinant human TWEAK (rTWEAK; Peprotech), (iii) 200 ng/mL Fn14 antagonist (ITEM‐4, sc‐56,250, Santa Cruz), and (iv) 100 ng/mL rTWEAK and 200 ng/mL ITEM‐4. ITEM4 has been utilized in previous studies as a neutralizing antibody targeting Fn14 to block TWEAK‐mediated effects in vitro [24, 25, 26]. Invasion assays were conducted similarly with Matrigel‐precoated chambers (CytoSelect 24‐Well Cell Invasion Assay Cell Biolabs Inc.) incubated at 37°C for 1 h. After 24 h, cells passing through the membrane were stained with 0.5% crystal violet and counted in five randomly selected high‐power fields (x200) under a microscope. All experiments were conducted three times.

### 2.8 Wound‐Healing Assay

Cells were cultured using a Culture‐Insert 2 Well in μ‐Dish 35 mm (ibidi GmbH) until reaching 80% confluence. The medium was replaced with a serum‐free medium, and the cells were cultured for 24 h. After the silicone insert removal, the medium was changed to 2% FBS medium containing the following conditions: (i) phosphate‐buffered saline control, (ii) 100 ng/mL rTWEAK, (iii) 200 ng/mL ITEM‐4, and (iv) 100 ng/mL rTWEAK and 200 ng/mL ITEM‐4. The wound area at 24 h was quantified using a microscope, and the percentage of wound closure was calculated using ImageJ software. All experiments were conducted three times.

### 2.9 Western Blotting

Proteins were extracted from cultured cells using RIPA buffer. The homogenates were centrifuged at 8000 revolutions/min. The protein concentrations of each sample were determined using a BCA protein assay kit (Thermo Fisher Scientific). Samples containing equal amounts of protein were resuspended in Laemmli Sample Buffer (Bio‐Rad Laboratories Inc.) containing 5% 2‐mercaptoethanol, separated by electrophoresis on 10% XV PANTERA Gels (NXV‐224P; DRC), and transferred to polyvinylidene difluoride membranes. Membranes were blocked in 5% skim milk for 1 h and then incubated overnight at 4°C with the primary antibodies shown in Table S1. After incubation with secondary antibodies at room temperature for 1 h, the membranes were developed using an enhanced chemiluminescence detection reagent (Nacalai Tesque Inc.) and visualized on a LAS‐4000UV mini luminescent image analyzer (FUJIFILM Wako Pure Chemical Corporation). The band intensities were quantified using ImageJ and normalized to β‐actin.

### 2.10 Statistical Analyses

Interobserver agreement for IHC staining was assessed using Cohen's kappa coefficient. Disease‐free survival (DFS) and overall survival (OS) were analyzed using the Kaplan–Meier method, and significance was examined with the log‐rank test. Multivariate analysis was performed using the Cox proportional hazards model. All potentially clinically important variables and high TWEAK/Fn14 expression were included in the multivariate analysis, regardless of their univariable *p*‐values. Categorical outcomes were compared using χ^2^ test or Fisher's exact test, and continuous outcomes were compared using Student's *t*‐test or Wilcoxon signed‐rank test. In vitro, all data are expressed as means and standard deviation. Data were analyzed with Student's *t*‐test. The data were analyzed using the JUMP software. All the tests were two‐tailed, and the results were considered significant at *p* < 0.05.

## 3 Results

### 3.1 Pathological Characteristics of CRLMs With rHGP

**FIGURE 2:**
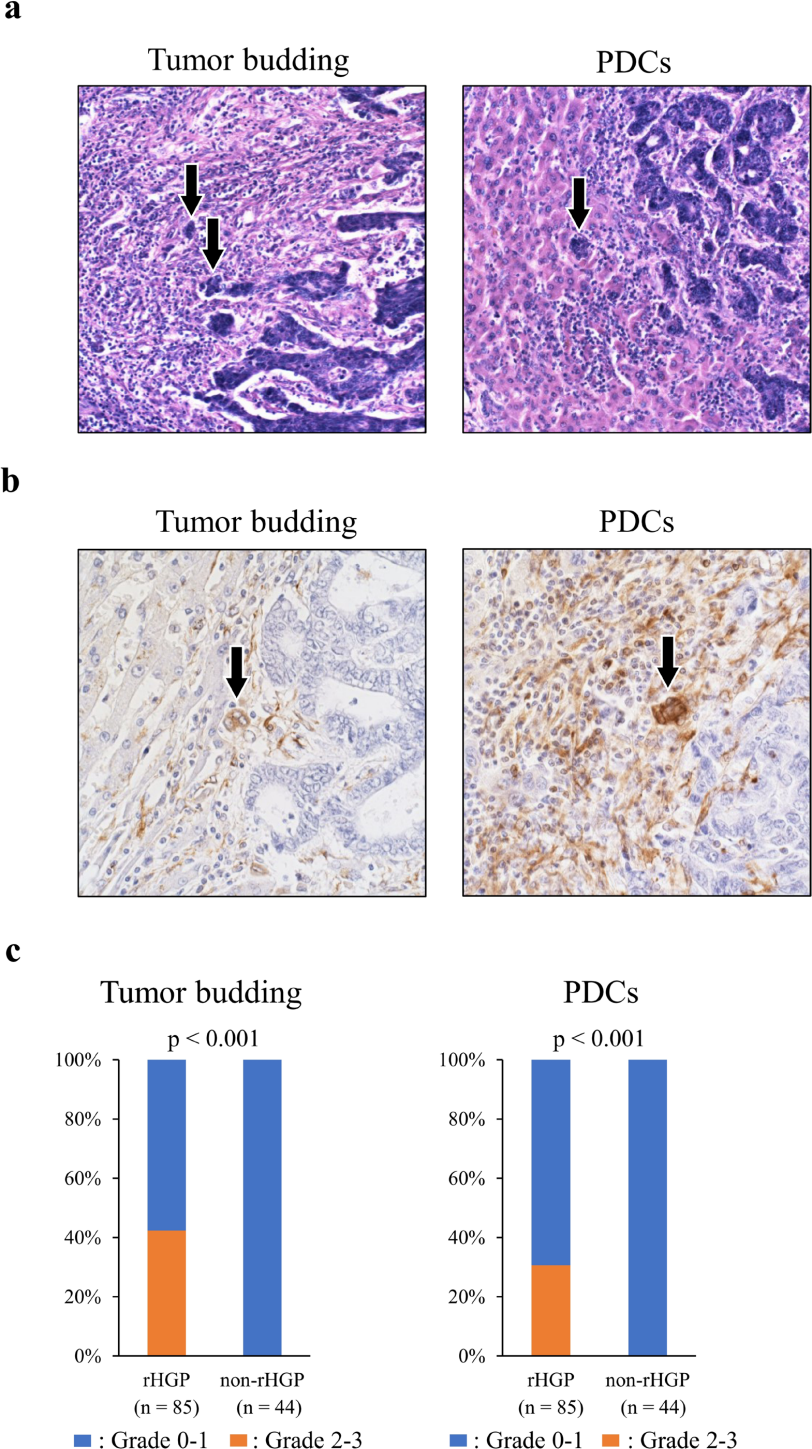
(a) Representative image of H&E staining of tumor budding (TB) and poorly differentiated clusters (PDCs) in CRLMs (black arrowhead). (b) Immunohistochemical staining for vimentin. Results are representative images of vimentin expression in TB and PDCs (black arrowhead). (c) CRLMs with replacement growth patterns were correlated with high grades of TB and PDCs.

To clarify the phenomenon occurring within CRLMs that affects tumor progression, pathological characteristics of HGPs in CRLM were initially investigated. Among the 129 patients with CRLMs who underwent initial liver resection, 31 were classified as having dHGP, 85 as rHGP, 5 as pHGP, and 8 as mixed HGP. The Kaplan–Meier analysis showed that patients with rHGP had significantly worse OS than those with other HGPs (non‐rHGP) (Figure 1b). The results suggested that the mechanism underlying rHGP may be crucial for tumor progression in CRLMs. To identify the pathological features at the invasive front of CRLMs depending on HGPs, TB and PDCs of CRLMs were examined (Figure 2a). As shown in Figure 2b, tumor cells in TB and PDCs of CRLMs showed vimentin expression immunohistochemically, suggesting EMT activation, which enhances metastases. Indeed, G2‐3 of TB cases and G2‐3 of PDCs cases experienced a significantly high frequency of extrahepatic metastases after surgery for CRLMs (Figure S1). Then, the relationship between HGPs and TB or PDCs was analyzed. CRLMs with rHGP exhibited a higher grade of TB and PDCs than CRLMs with non‐rHGP (Figure 2c). These results indicate that CRLMs of rHGP have EMT activation and a high metastatic potential.

### 3.2 Tumor Microenvironment of CRLM With rHGP

**FIGURE 3:**
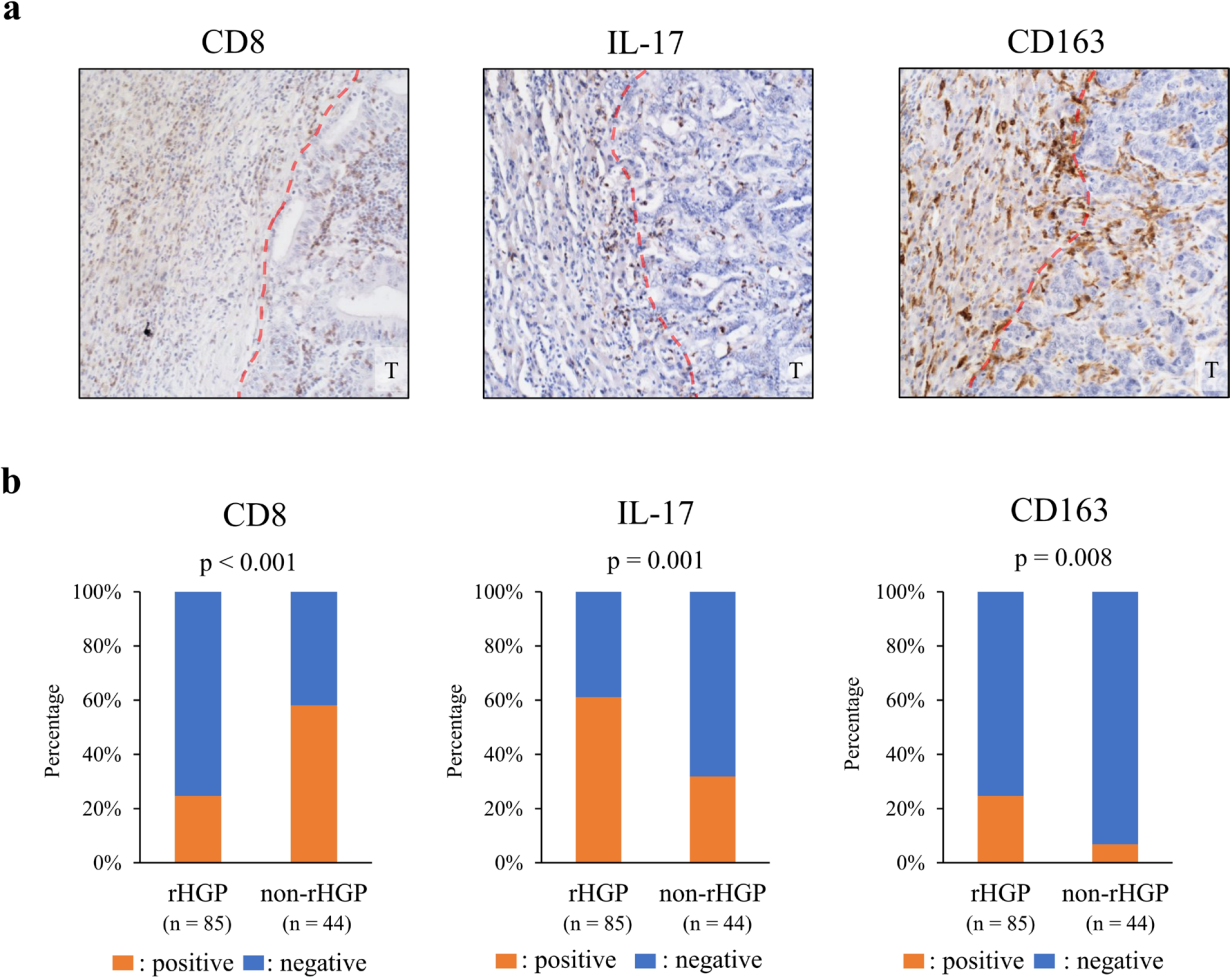
(a) Immunohistochemical staining for CD8, IL‐17, and CD163 in CRLMs (T: Tumor). CRLMs with immune cell infiltration (CD8, IL‐17, and CD163) in the tumor at the invasive front were defined as positive. (b) CRLMs with replacement growth pattern (rHGP) were negatively correlated with CD8 expression, whereas CRLMs with rHGP were positively correlated with IL‐17 and CD163 expressions.

The tumor microenvironment in CRLMs has been reported to play an important role in tumor progression [27, 28]. To assess the specific infiltration of inflammatory cells in CRLMs with rHGP, the distributions of CD8‐positive cells, IL‐17‐positive cells, and CD163‐positive cells at the invasive front of CRLMs were investigated immunohistochemically (Figure 3a). Cases with CD8 positivity had significantly better OS than those with CD8 negativity, whereas cases with IL‐17 positivity and those with CD163 positivity had significantly worse OS than other cases (Figure S2). CD8 staining also revealed significantly less CD8‐T cell infiltration in CRLMs with rHGP than CRLMs with non‐rHGP, whereas IL‐17 and CD163 staining revealed that CRLMs with rHGP had more infiltration of Th17 cells and CD163‐positive macrophages than CRLMs with non‐rHGP (Figure 3b). These data suggested that CRLMs with rHGP have a specific tumor microenvironment mediated by Th17 cells and CD163‐positive macrophages.

### 3.3 Expression of TWEAK and Fn14 in CRLM

**FIGURE 4:**
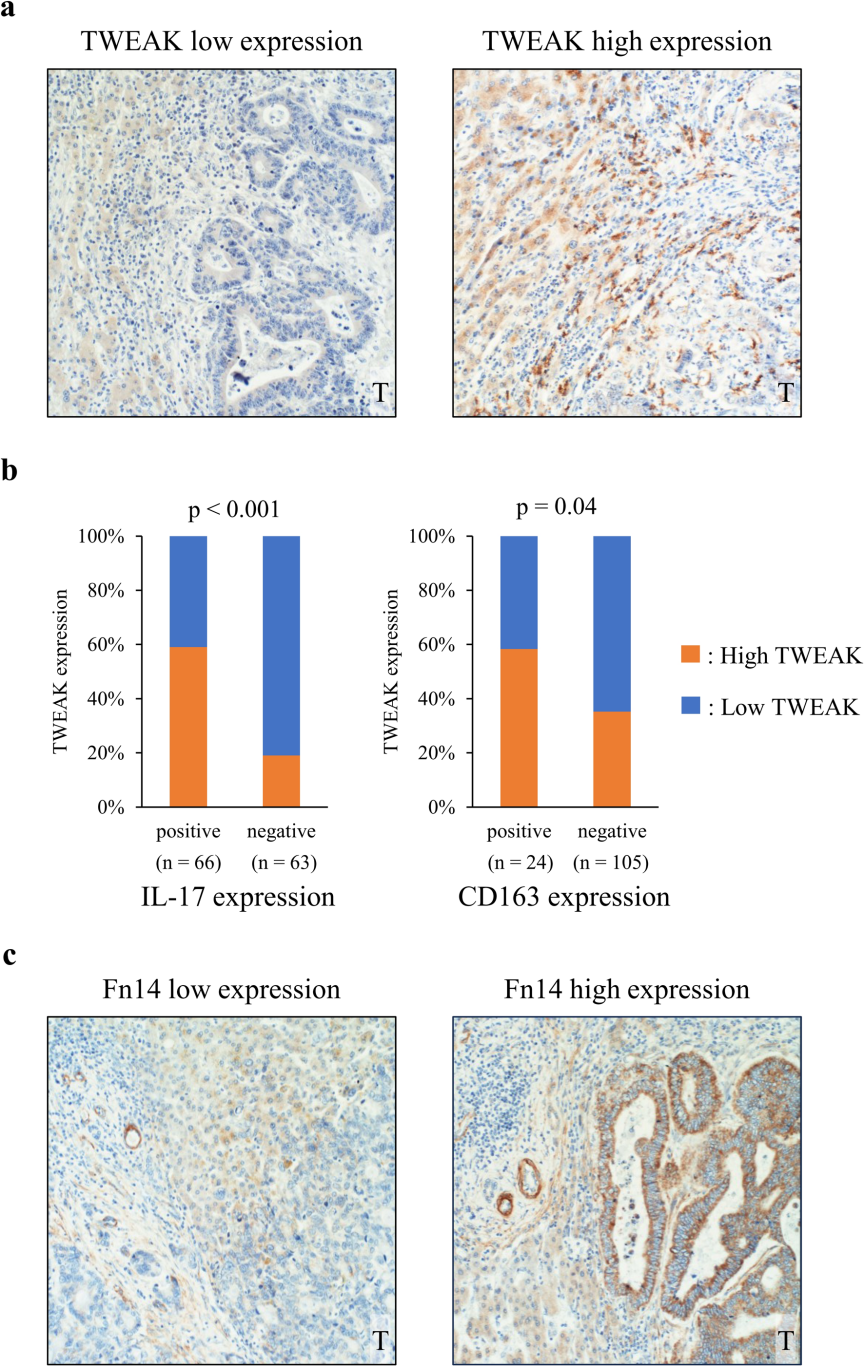
(a) Immunohistochemical staining for TWEAK in CRLMs. CRLMs with TWEAK expression in the cytoplasm of immune cells and stroma surrounding the tumor margin of CRLMs were defined as high TWEAK expression (T: Tumor). (b) CRLMs with high TWEAK expression were positively correlated with IL‐17 and CD163 expression. (c) Immunohistochemical staining for Fn14 in CRLMs. CRLMs with Fn14 expression in the cell membrane and cytoplasm of tumor cells were defined as high Fn14 expression.

**FIGURE 5:**
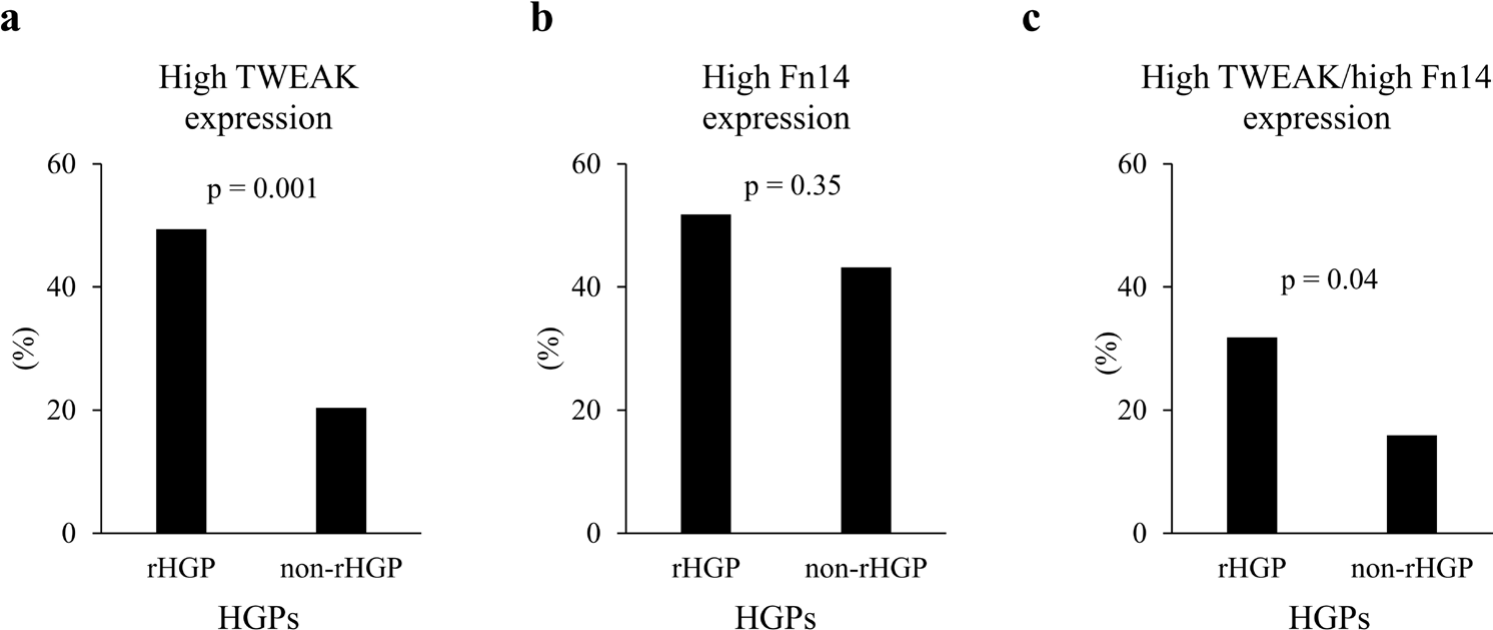
(a) CRLMs with replacement growth pattern (rHGP) exhibited significantly higher TWEAK expression than CRLMs with non‐rHGP. (b) There was no significant difference between rHGP and Fn14 expression. (c) High TWEAK/high Fn14 expression was observed more frequently in CRLMs with rHGP than in CRLMs with non‐rHGP.

Our data indicated that CRLMs with rHGP, a phenotype associated with poor prognosis, exhibit EMT activation concomitant with the infiltration of Th17 cells and CD163‐positive macrophages. To clarify the mechanism by which the tumor microenvironment affects tumor progression in CRLMs with rHGP, the significance of the TWEAK/Fn14 axis in CRLMs was investigated. Initially, the expression of TWEAK and Fn14 in CRLM was examined. Immunohistochemical staining revealed that TWEAK was expressed in the cytoplasm of immune cells and stroma surrounding the tumor margin in CRLMs (Figure 4a). Minimal expression of TWEAK was observed in cancer cells. Of the 129 patients, 51 (39.5%) showed high TWEAK expression at the invasive front of CRLMs (Interobserver agreement; κ = 0.83). High TWEAK expression was positively correlated with expression of IL‐17 and CD163 (Figure 4b). Immunohistochemical staining of serial sections for TWEAK, IL‐17, and CD163 showed that TWEAK expression was localized around IL‐17‐positive immune cells and CD163‐positive immune cells (Figure S3). These results proposed that Th17 cells and M2 macrophages may contribute to the release of TWEAK in CRLMs. In contrast, the immunohistochemical staining revealed that Fn14 was expressed in the cell membrane and cytoplasm of the tumor cells of CRLMs (Figure 4c), and high Fn14 expression in the tumor cells was detected in 63 (48.8%) patients (Interobserver agreement; κ = 0.84). Immunohistochemical staining for TWEAK and Fn14 by serial section revealed that TWEAK expression was observed surrounding Fn14‐positive cancer cells (Figure S4). Then, the correlation between TWEAK/Fn14 expression and HGPs was investigated. CRLMs with rHGP exhibited significantly higher TWEAK expression compared to those with non‐rHGP (Figure 5a). In contrast, while CRLMs with rHGP showed a higher rate of high Fn14 expression, the difference was not statistically significant (Figure 5b). Consequently, cases with both high TWEAK and Fn14 expression (high TWEAK/high Fn14) were significantly more frequent in CRLMs with rHGP than other HGPs (Figure 5c). These results indicated that the TWEAK/Fn14 axis may be activated specifically in the tumor microenvironment of CRLMs with rHGP.

### 3.4 Effect of the TWEAK/Fn14 Axis on Patient Survival and Tumor Progression in CRLMs

**FIGURE 6:**
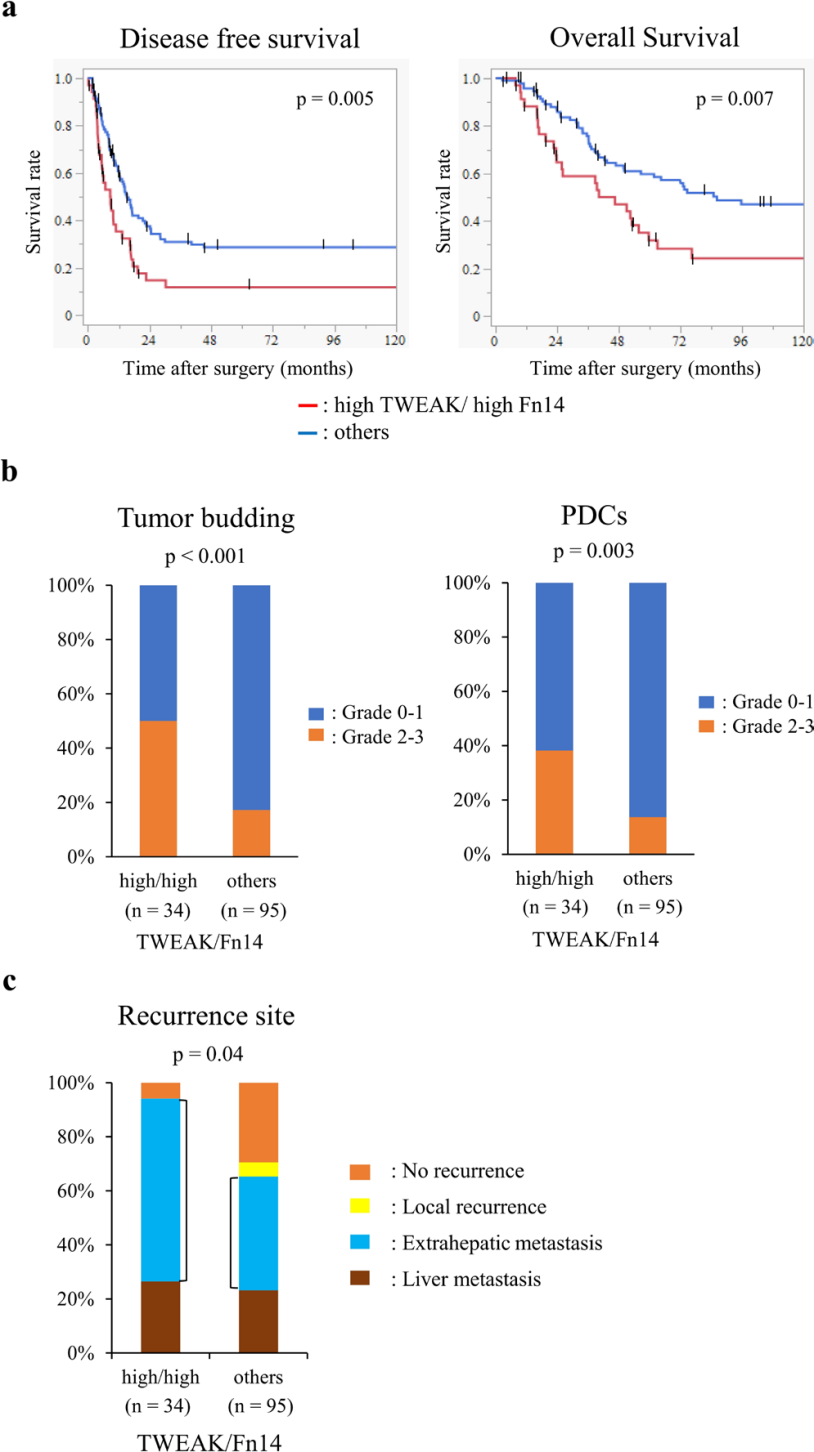
(a) Disease‐free and overall survival after liver resection in the high TWEAK/high Fn14 group versus other groups was analyzed by the Kaplan–Meier method. (b) High TWEAK/high Fn14 expression was correlated with grades of tumor budding and poorly differentiated clusters (PDCs). (c) High TWEAK/high Fn14 group had a significantly higher frequency of extrahepatic metastases.

Then, the effects of the TWEAK/Fn14 axis on patient prognosis and tumor progression in CRLMs were clarified. To determine whether the TWEAK/Fn14 axis in CRLMs is associated with clinicopathological features, the characteristics of patients with high TWEAK/high Fn14 (*n* = 34) are summarized in Table 1. No significant difference was found in clinicopathological factors, except for sex, between patients with high TWEAK/high Fn14 and other patients. Then, whether the TWEAK/Fn14 axis in CRLMs is related to the prognosis after liver resection was investigated using the Kaplan–Meier method. The high TWEAK/high Fn14 group had significantly poorer DFS and OS than the other groups (Figure 6a). Univariate and multivariate analyses were also performed to investigate whether the TWEAK/Fn14 axis is an independent OS factor. The multivariate analysis identified lymph node metastases of the primary lesion, concomitant extrahepatic metastases, high TWEAK/high Fn14 expression, and adjuvant chemotherapy as independent prognostic factors for OS after liver resection (Table 2). The multivariate analysis for DFS also identified high TWEAK/high Fn14 expression as an independent prognostic factor for DFS after liver resection (Table S2). Finally, the effect of the TWEAK/Fn14 axis on tumor progression in CRLMs was examined. The high TWEAK/high Fn14 group exhibited TB and PDCs significantly more frequently than the other groups (Figure 6b). These results indicated that the TWEAK/Fn14 axis in CRLMs is associated with poor prognosis and is accompanied by TB and PDCs, which are indicative of EMT activation. In addition, the high TWEAK/high Fn14 group had significantly higher rates of extrahepatic recurrence after liver resection (Figure 6c).

**TABLE 1:** Associations between patients with colorectal liver metastases expressing both high TWEAK and high Fn14 and clinicopathological features.

Factors		High TWEAK/high Fn14 (*n* = 34)	Others (*n* = 95)	*p*
Age	< 60	11 (31%)	24 (69%)	0.43
	60 ≤	23 (24%)	71 (76%)	
Sex	Male	12 (15%)	60 (85%)	0.005
	Female	22 (39%)	35 (61%)	
Timing of metastases	Synchronous	21 (31%)	47 (69%)	0.22
	Metachronous	13 (21%)	48 (79%)	
Primary lesion site	Colon	20 (26%)	58 (74%)	0.82
	Rectum	14 (27%)	37 (73%)	
Lymph node metastases of primary lesion	N (−)	10 (22%)	36 (78%)	0.35
	N (+)	24 (29%)	58 (71%)	
Tumor size	< 50 mm	25 (27%)	69 (73%)	0.92
	50 mm ≤	9 (26%)	26 (74%)	
Number of tumors	< 5	25 (25%)	76 (27%)	0.44
	5 ≤	9 (32%)	19 (68%)	
Extrahepatic metastases	(−)	25 (24%)	79 (76%)	0.23
	(+)	9 (36%)	16 (64%)	

**TABLE 2:** Univariate and multivariate analysis for overall survival in patients with colorectal liver metastases.

Factors		Univariate analysis			Multivariate analysis		
		Hazard ratio	95% CI	*p*	Hazard ratio	95% CI	*p*
Age	< 60	1.56	0.96–2.55	0.07			
	60 ≤						
Sex	Female	1.10	0.69–1.75	0.70			
	Male						
Timing of metastases	Synchronous	1.25	0.78–2.01	0.35	1.15	0.67–1.97	0.60
	Metachronous						
Primary lesion site	Rectum	1.23	0.77–1.98	0.39			
	Colon						
Lymph node metastases of primary lesion	N (+)	1.83	1.07–3.13	0.03	2.23	1.21–4.10	0.01
	N (−)						
Tumor size (mm)	50 mm ≤	1.54	0.93–2.55	0.09	1.43	0.83–2.47	0.19
	< 50 mm						
Number of tumors	5 ≤	1.57	0.93–2.66	0.09	1.20	0.66–2.18	0.55
	< 5						
Extrahepatic metastases	(+)	2.17	1.28–3.68	< 0.01	2.55	1.35–4.82	< 0.01
	(−)						
TWEAK/Fn14	high/high	1.95	1.19–3.19	< 0.01	1.81	1.10–2.98	0.02
	others						
Adjuvant chemotherapy	(−)	1.64	1.01–2.69	0.048	1.73	1.03–2.90	0.04
	(+)						

### 3.5 Effect of the TWEAK/Fn14 Axis on CRC Cell Lines

**FIGURE 7:**
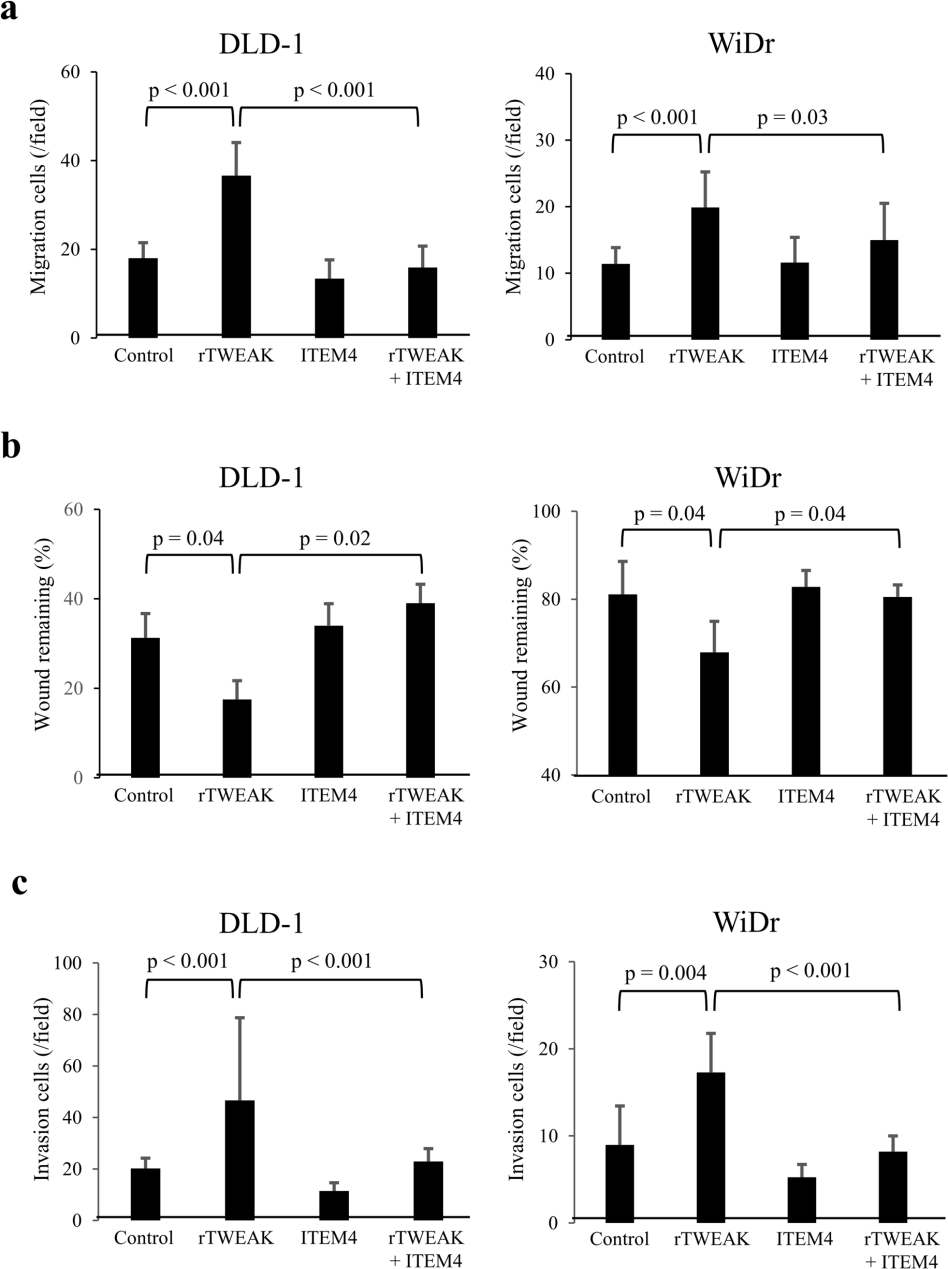
(a) The effects of recombinant TWEAK (rTWEAK) on the migration of colorectal cancer (CRC) cells and its inhibition by Fn14 antagonist (ITEM‐4) were evaluated by migration assay (*n* = 5 per group). Cells passing through the membrane were counted in five randomly selected high‐power fields (x200) under a microscope. (b) The effects of rTWEAK on the migration capability of CRC cells and its inhibition by ITEM‐4 were evaluated by wound‐healing assay. The remaining wound area was measured under a microscope (*n* = 4 per group). (c) The effects of rTWEAK on the invasive ability of CRC cells and its inhibition by ITEM‐4 were evaluated by invasion assay (*n* = 5 per group). Cells passing through the membrane were counted in five randomly selected high‐power fields (x200) under a microscope. Data are expressed as means ± standard deviation.

On the basis of the clinical findings indicating the association of TWEAK/Fn14 expression with aggressive tumor biology in CRLMs, in vitro experiments were performed to examine the effects of the TWEAK/Fn14 axis on tumor progression using two CRC cell lines, DLD‐1 and WiDr. First, Fn14 expression was detected in both cell lines through Western blot analysis (Figure S5). The cells were then divided into a control group and a rTWEAK group (100 ng/mL) and cultured for 24, 48, and 96 h for the proliferation assay. No significant difference in cell proliferation was found between the control group and the rTWEAK group in both DLD‐1 and WiDr (Figure S6). To investigate whether TWEAK/Fn14 influences the migratory ability of CRC cells, a migration assay and a wound healing assay were conducted. In the migration assay, the number of cells passing through the membrane increased significantly in both DLD‐1 and WiDr cells with rTWEAK exposure (Figure 7a). The addition of ITEM‐4 inhibited the increase in migratory cells induced by rTWEAK, whereas ITEM‐4 exposure did not affect the number of migratory cells (Figure 7a). These results indicate that rTWEAK increases cell migration through Fn14. Similarly, in a wound healing assay, a significant increase in the gap closure rate was observed in both DLD‐1 and WiDr cells with rTWEAK exposure, and this effect was suppressed by ITEM‐4 (Figure 7b). To investigate whether TWEAK/Fn14 influences the invasiveness of CRC cells, an invasion assay was conducted. In an invasion assay using a Matrigel membrane, a significant increase in the number of cells passing through the membrane was observed with rTWEAK exposure in both DLD‐1 and WiDr cells, which was not observed with rTWEAK plus ITEM‐4 (Figure 7c). These findings suggest that the TWEAK/Fn14 axis promotes migration and invasion in CRC cells.

### 3.6 Activated Signaling Pathway and Alteration of EMT Markers in Response to rTWEAK

**FIGURE 8:**
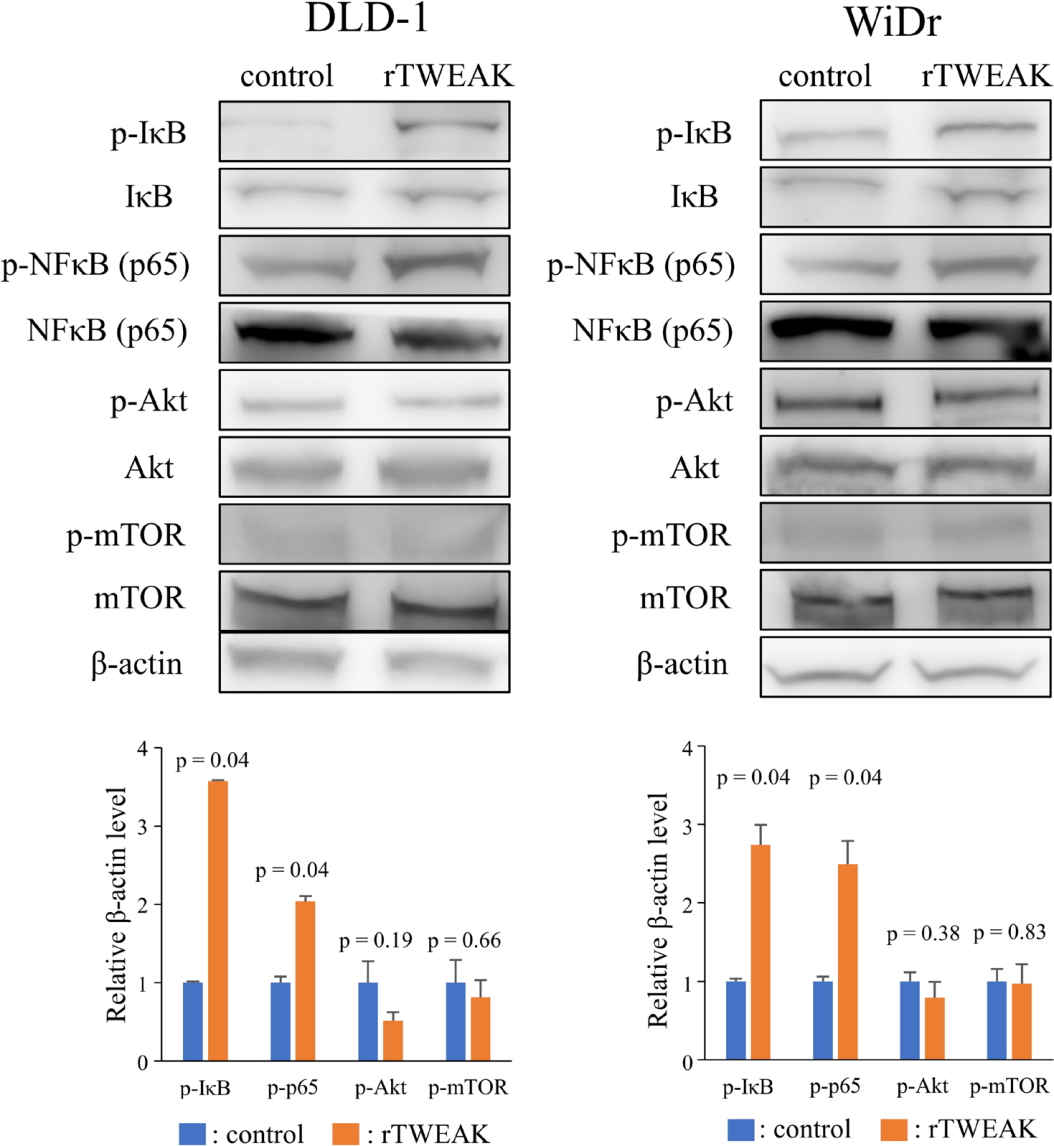
Activation of NFκB pathway and Akt–mTOR pathway mediated by recombinant TWEAK (rTWEAK) in colorectal cancer cells was evaluated by Western blotting (*n* = 3 per group). Data are expressed as means ± standard deviation.

**FIGURE 9:**
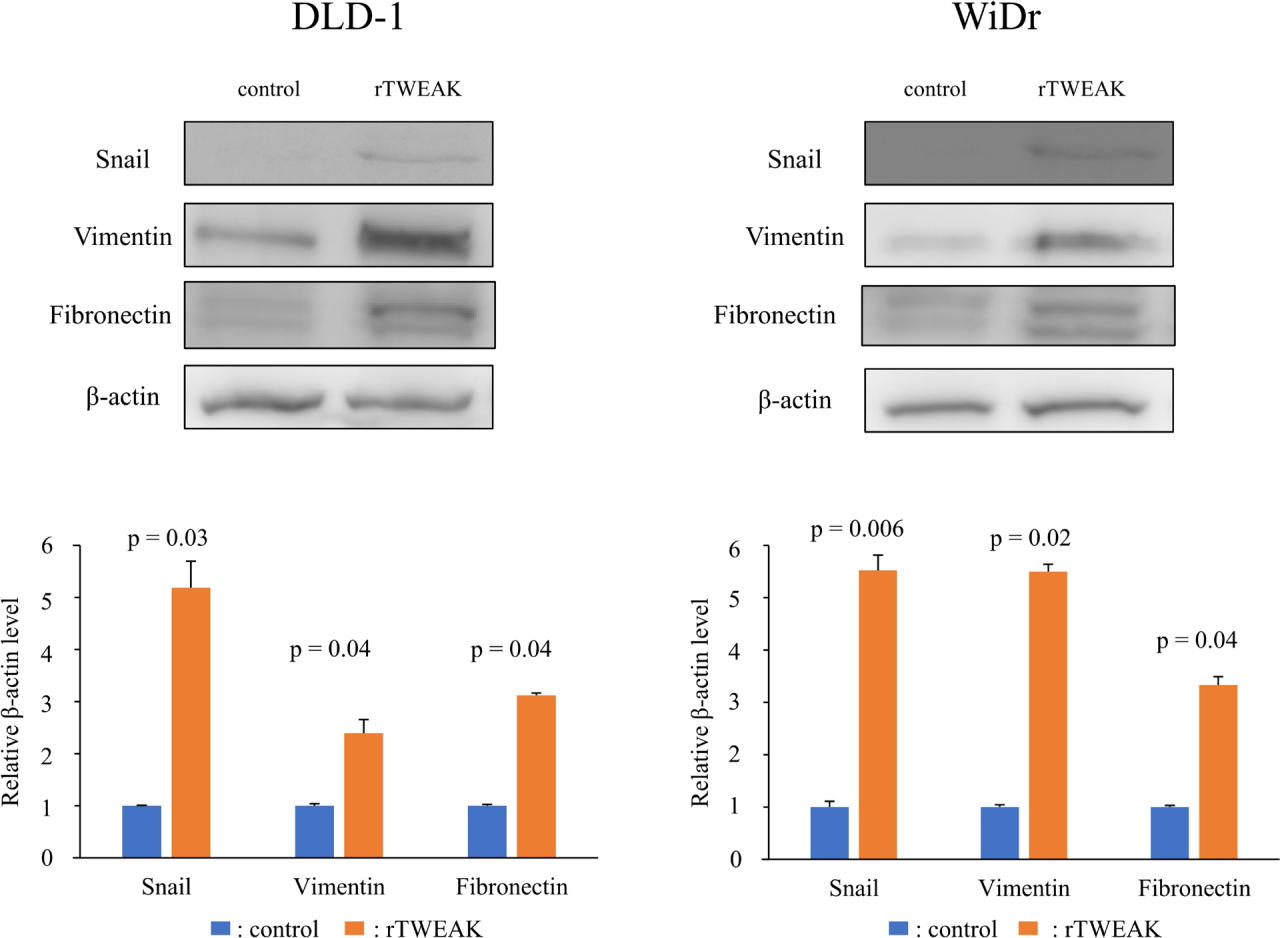
The effects of recombinant TWEAK (rTWEAK) on EMT marker expression in colorectal cancer cells were evaluated by Western blotting (*n* = 3 per group). Data are expressed as means ± standard deviation.

Previous reports showed that the TWEAK/Fn14 axis mainly regulates the NFκB pathway or Akt–mTOR pathway [13, 29]. To clarify the intracellular signaling pathway activated by the TWEAK/Fn14 axis in CRC cells, Western blot analyses for NFκB pathway and Akt–mTOR pathway were performed. rTWEAK significantly induced phosphorylation of IκB and NFκB (p65) in both DLD‐1 and WiDr cells, whereas no increase in phosphorylated Akt and mTOR was observed by rTWEAK exposure (Figure 8). Furthermore, in rTWEAK‐treated CRC cells, Western blot analyses revealed a significant increase in the expression of mesenchymal markers, such as snail, vimentin, and fibronectin (Figure 9). These findings suggest that the TWEAK/Fn14 axis may activate the canonical NFκB pathway and promote EMT in CRC cells.

## 4 Discussion

This study investigated the effects of the TWEAK/Fn14 axis in CRLMs on tumor progression. TWEAK was secreted at the invasive front of CRLMs with the infiltration of Th17 cells and M2 macrophages, whereas Fn14 was expressed in tumor cells surrounding TWEAK. The TWEAK/Fn14 axis in CRLMs, specifically observed in rHGP, is correlated with TB and PDCs, which may contribute to extrahepatic metastases. In addition, the TWEAK/Fn14 axis in CRLMs was an independent factor that affects poor survival. In vitro, rTWEAK enhances tumor migration and invasion via Fn14 in CRC cell lines. This study suggests that the TWEAK/Fn14 axis in the tumor microenvironment of CRLMs plays a crucial role in tumor progression, particularly in rHGP, which is a major CRLM phenotype associated with poor prognosis.

The ‘Seed and soil’ hypothesis suggests that the metastatic environment determines the development and progression of tumor metastases [30]. The interaction between the liver, as the host, and metastatic tumor cells may influence the HGPs of CRLMs [31, 32], and our data confirmed that rHGP is a pathological feature of poor prognosis. The underlying mechanism by which patients with rHGP are associated with a poor prognosis is not well understood. Previous studies have shown that CRLMs of rHGP do not have a high proliferative potential compared with CRLMs of dHGP [33, 34]. On the contrary, this study identified the EMT status of metastatic tumor cells as a key phenomenon in the tumor progression of CRLMs with rHGP. High grade of TB and PDCs were detected in CRLMs with rHGP. As previously described in primary CRC [35], this study demonstrated that TB and PDCs in CRLMs also exhibit the EMT state, as confirmed by vimentin expression, and patients with TB and PDCs in CRLMs had higher tumor recurrence rates, particularly extrahepatic metastases. These results indicate that CRLMs with rHGP have high invasive and metastatic potential, resulting in a poor prognosis after liver resection for CRLMs. This study also identified specific immune microenvironment in CRLMs with rHGP. Low CD8‐T cell infiltration in CRLMs with rHGP suggested minimal anti‐tumor microenvironment in rHGP. Conversely, prominent infiltration of Th17 cells and M2 macrophages was observed in CRLMs with rHGP. Th17 cells could promote the expansion of regulatory T cells through the release of IL‐17A, facilitating immune evasion by tumor cells [36]. M2 macrophages establish an immunosuppressive tumor microenvironment and trigger the metastatic cascade of tumor cells [37, 38]. These cells infiltrating the CRLMs with rHGP may play a role in tumor progression, as evidenced by their association with poor prognosis shown in our data. The rationale of this study was to explore the biological mechanisms by which the tumor microenvironment in rHGP influences tumor invasion and metastatic potential in CRLMs.

The TWEAK/Fn14 axis has been reported to promote cell proliferation, invasion, differentiation, and apoptosis in various cancers [16, 17]. TWEAK and Fn14 expression have been identified in primary CRC [16, 39, 40]; however, their expression and roles in CRLMs have not been evaluated. The present study revealed that TWEAK expression was localized in the immune cells and the stroma surrounding the tumor margin of CRLMs, which was correlated with the infiltration of Th17 cells and CD163‐positive macrophages. Previous studies have demonstrated that Th17 cells and tumor‐associated macrophages can secrete TWEAK in the tumor microenvironment [15, 19]. TWEAK expression in primary CRC cells has been identified previously; however, its expression is lost in advanced CRC [18]. On the basis of our findings, TWEAK expression in the metastatic tumor cells may be minimal, and its secretion in CRLMs may be mainly mediated by inflammatory cells, such as Th17 cells and CD163‐positive macrophages, in the tumor microenvironment. Our data also demonstrated that TWEAK expression was frequently observed in CRLMs with rHGP, and high TWEAK/high Fn14 cases were prevalent in rHGP. These results suggest that the TWEAK/Fn14 axis may regulate tumor progression in the tumor microenvironment of CRLMs with rHGP. Previous studies have demonstrated that high Fn14 expression serves as a prognostic factor in several cancers [41, 42, 43]. However, Fn14 expression in CRLMs alone was not associated with a poor prognosis (data not shown). There was a spatial proximity of TWEAK and Fn14 at the invasive front of CRLMs, and patients with both high TWEAK and high Fn14 expression was an independent factor that affects poor survival in the multivariate analyses. These results indicate that the TWEAK‐mediated activation of Fn14 may lead to poor survival in patients with CRLMs.

A recent study revealed that the TWEAK/Fn14 axis in primary CRC contributes to the development of metastases from the primary tumor [19]. In contrast, our data showed that both high TWEAK and high Fn14 expression in CRLMs were not associated with the timing of metastases, the number of CRLMs, and the size of CRLMs, indicating that their expression in CRLMs may not relate to CRLM development from the primary lesion. There may be different tumor microenvironments between primary CRC and CRLMs, as shown previously [44, 45]. On the other hand, CRLMs with high TWEAK/high Fn14 expression exhibited a high frequency of TB and PDCs, which was concomitant with the high recurrence risk of extrahepatic metastases. These results suggest that the TWEAK/Fn14 axis in the tumor microenvironment of CRLMs enhances the invasive and metastatic potential of tumor cells in CRLMs. In vitro experiments supported these clinical findings by demonstrating that rTWEAK significantly enhanced cell chemotaxis and invasion without affecting cell proliferation in CRC cell lines. rTWEAK increased the expression of EMT markers such as snail, vimentin, and fibronectin. TWEAK may promote EMT in CRC cells by enhancing snail expression. Notably, the enhanced motility and invasiveness induced by rTWEAK were reduced by an Fn14 antagonist, indicating that TWEAK promotes tumor progression via Fn14. The results also suggest that targeting Fn14 could serve as a promising therapeutic strategy to mitigate EMT and reduce the metastatic spread in CRLMs. Regarding the genetic profiles of colorectal cancer cell lines used in this study, DLD‐1 harbors a KRAS G13D mutation, and WiDr harbors a BRAF V600E mutation [46]. A recently published study reported that TWEAK enhanced tumor invasion in the SW48 colorectal cell line, which is wild‐type for both RAS and BRAF [19]. Together, these findings suggest that the TWEAK/Fn14 axis promotes tumor progression and may represent a therapeutic target independent of RAS and BRAF mutation status. In addition, our study focused on the intracellular signaling pathway activated by the TWEAK/Fn14 axis in CRC cells. NFκB pathway enhanced EMT in CRC [47], whereas phosphorylated mTOR induces proliferation of CRC [46]. Furthermore, a previous study demonstrated that activation of NFκB pathway stabilizes Snail expression, which in turn enhances tumor invasion and migration in cancer cell lines [48]. Our data indicated that the TWEAK/Fn14 axis may activate the canonical NFκB pathway and promote EMT and cellular invasiveness.

This study has some limitations. First, the clinical findings were on the basis of the retrospective nature of data collection with a relatively small sample size. However, this is the first study to clearly describe the role of the TWEAK/Fn14 axis in CRLMs with rHGP and the effect of the TWEAK/Fn14 expression in CRLMs on patient survival. Second, the effect of the TWEAK/Fn14 axis on migration and invasion was demonstrated only by an in vitro study using human CRC cell lines. Further studies utilizing CRLM‐specific in vivo models are necessary to validate these findings. Third, this study did not assess the expression of TWEAK and Fn14 in primary colorectal cancer. Although the concordance of TWEAK and Fn14 expression between CRLMs and matched primary tumors remains undetermined, our study identified upregulated TWEAK and Fn14 expression in CRLMs, which suggests that the TWEAK/Fn14 axis may serve as a therapeutic target in metastatic colorectal cancer.

## 5 Conclusions

The TWEAK/Fn14 axis in the tumor microenvironment of CRLMs may worsen patient prognosis by enhancing the migratory and invasive ability of metastatic tumor cells. The TWEAK/Fn14 axis represents a key molecular mechanism underlying the poor prognosis of rHGP.

## Author Contributions

**Naoki Matsuyama:** methodology (lead); writing – original draft (equal); formal analysis (lead); Investigation (lead). **Takanori Konishi:** conceptualization (lead); writing – original draft (equal); formal analysis (supporting); investigation (supporting), funding Acquisition (lead). **Tsukasa Takayashiki:** writing – review and editing (equal). Shigetsugu Takano: Writing – review and editing (equal). Daisuke Suzuki: Writing – review and editing (equal). Nozomu Sakai: Writing – review and editing (equal). **Isamu Hosokawa:** writing – review and editing (equal). **Takashi Mishima:** formal analysis (supporting); resources (lead); investigation (supporting). **Kensuke Suzuki:** methodology (supporting). Hitoe Nishino: Writing – review and editing (equal). **Shinichiro Nakada:** writing – review and editing (equal). Masayuki Ohtsuka: Conceptualization (supporting); supervision (lead).

## Ethics Statement

The study was approved by the Committee on Human Research of Chiba University Hospital (Approval number: M10528).

## Consent

All patients provided a written informed consent according to the Declaration of Helsinki.

## Conflicts of Interest

The authors declare no conflicts of interest.

## Supporting information


**Figure S1.** Recurrence sites after liver resection depending on the grade of tumor budding or PDCs.
**Figure S2.** Influence of infiltrating immune cells within CRLMs on prognosis.
**Figure S3.** Immunohistochemical staining for TWEAK, IL‐17, and CD163 by serial sections.
**Figure S4.** Immunohistochemical staining for TWEAK and Fn14 by serial sections.
**Figure S5.** Fn14 expression in human colorectal cancer cell lines.
**Figure S6.** Effects of recombinant TWEAK on proliferation of colorectal cancer cells.

**Table S1.** List of primary antibodies used in this study.

**Table S2.** Univariate and multivariate analysis for disease free survival in patients with colorectal liver metastases.

## Data Availability

Data available on request from the authors.
