# Supplementary material for: TWEAK/Fn14 Drives Tumor Progression and Is Associated With Poor Survival of Colorectal Liver Metastases With Replacement Growth Patterns
Source: Cancer Med. 2025 Jul 9;14(13):e71027. doi: 10.1002/cam4.71027 (PMC12238726; doi:10.1002/cam4.71027)

**Fig. S1.** **Recurrence sites after liver resection depending on the grade of tumor budding or PDCs.**

G2-3 of tumor budding cases and G2-3 of poorly differentiated clusters (PDCs) cases experienced a significantly high frequency of extrahepatic metastases after surgery for CRLMs.

**
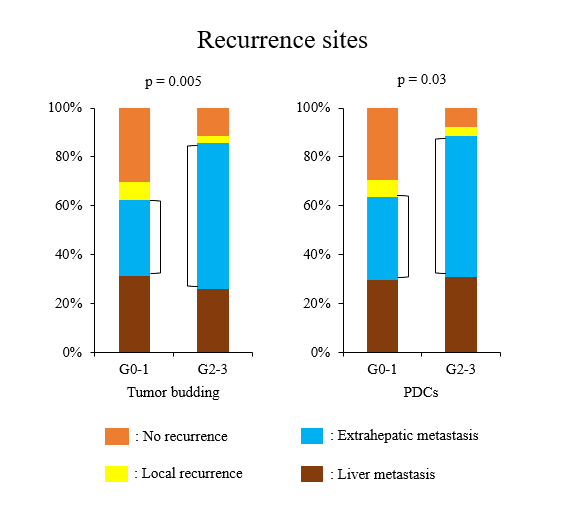
**

**Fig. S2. Influence of infiltrating immune cells within CRLMs on prognosis**

Overall survival after liver resection stratified with the infiltration of immune cells in CRLMs are analyzed using Kaplan-Meier method.


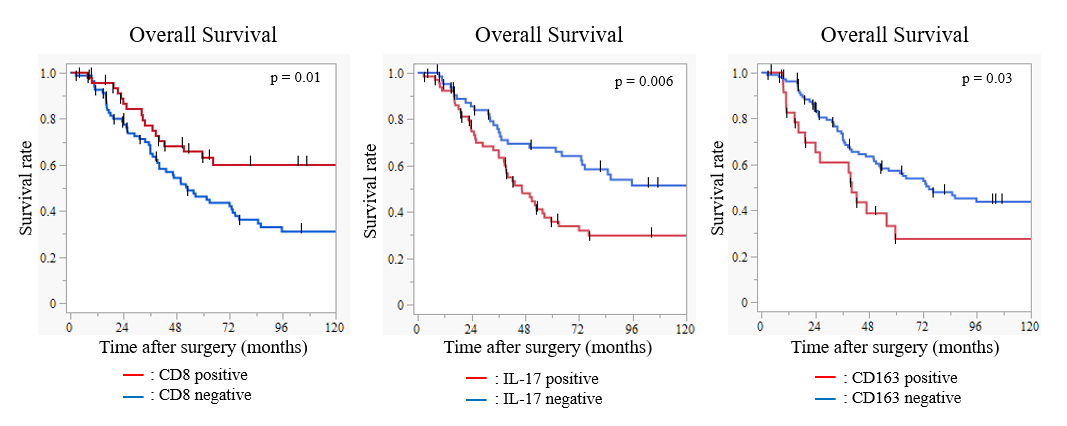


**Fig. S3. Immunohistochemical staining for TWEAK, IL-17, and CD163 by serial sections**

Results are representative images of the co-localization of TWEAK expression and IL-17-positive immune cells and CD163-positive immune cells. (T: tumor, N: normal liver)


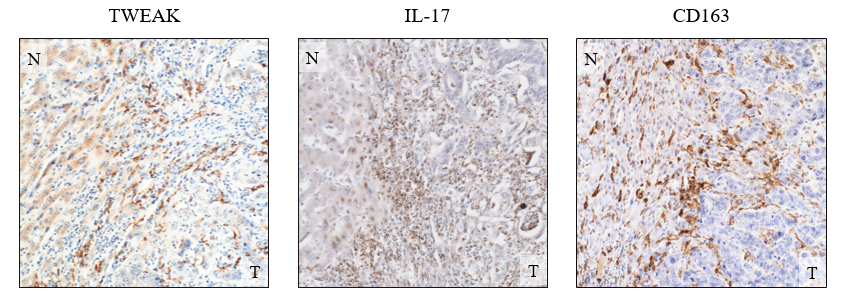


**Fig. S4.** **Immunohistochemical staining for TWEAK and Fn14 by serial sections**

Results are representative images of the co-localization of TWEAK-positive inflammatory cells and Fn14-positive cancer cells. TWEAK-positive cells and Fn14-positive cells are indicated by arrows.　(T:tumor)

**
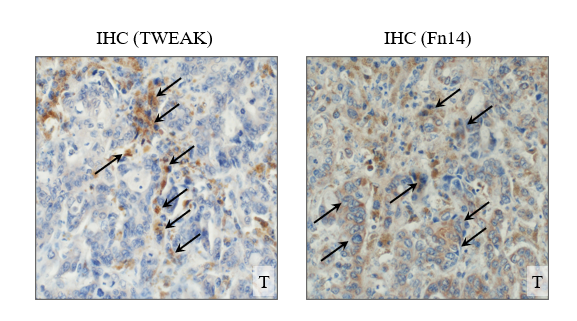
**

**Fig. S5.** **Fn14 expression in human colorectal cancer cell lines**

The expression of Fn14 in the human colorectal cancer cell lines DLD-1 and WiDr were evaluated by Western blotting.


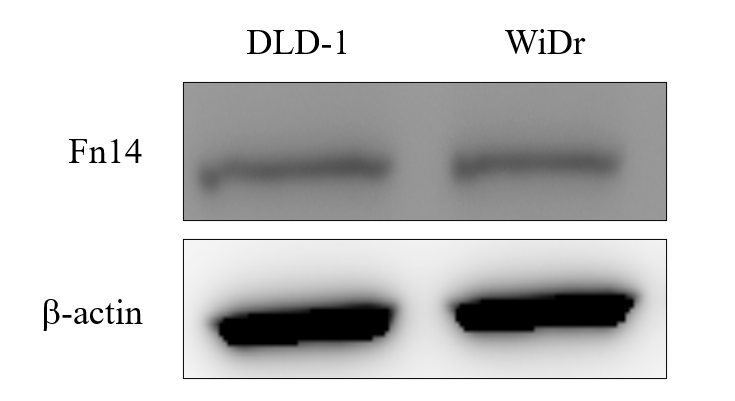


**Fig. S6.** **Effects of recombinant TWEAK on proliferation of colorectal cancer cells**

The effects of recombinant TWEAK (rTWEAK) on the proliferation of colorectal cancer cells were evaluated by proliferation assay (n = 10 per group).


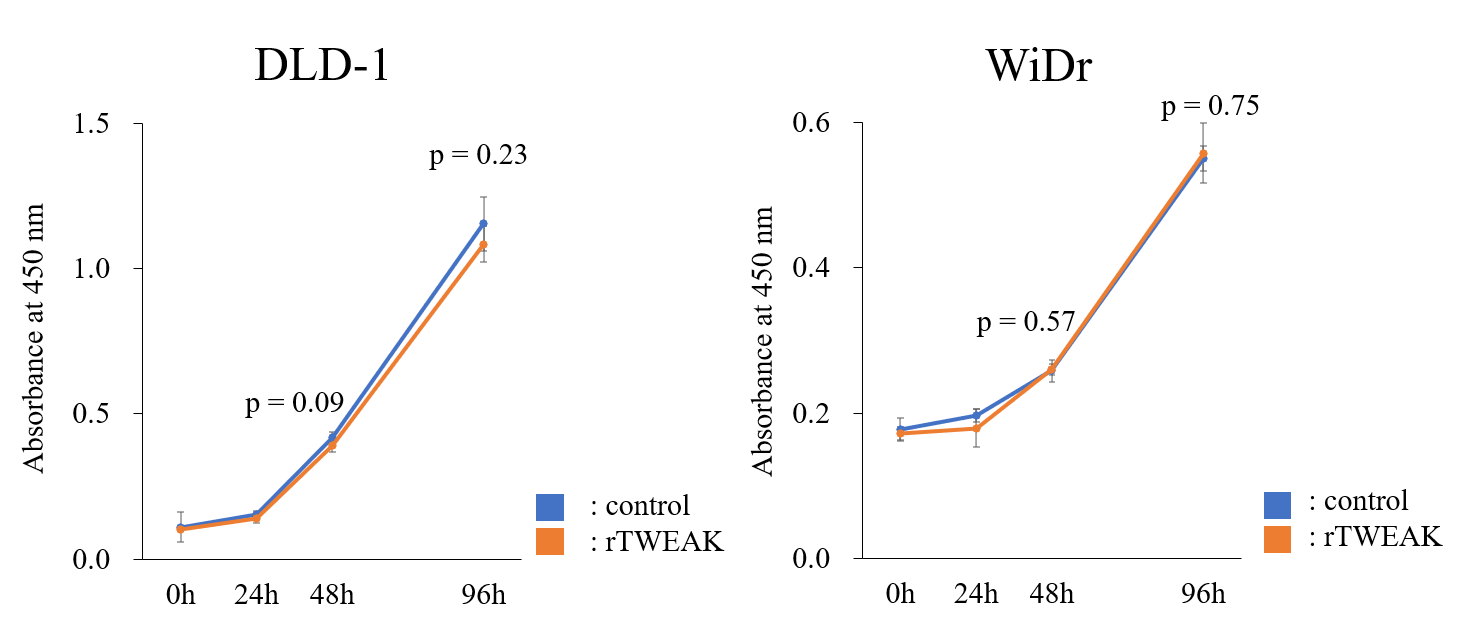

Supplement: Supplementary file 1 — Figure S1. Recurrence sites after liver resection depending on the grade of tumor budding or PDCs. Figure S2. Influence of infiltrating immune cells within CRLMs on prognosis. Figure S3. Immunohistochemical staining for TWEAK, IL‐17, and CD163 by serial sections. Figure S4. Immunohistochemical staining for TWEAK and Fn14 by serial sections. Figure S5. Fn14 expression in human colorectal cancer cell lines. Figure S6. Effects of recombinant TWEAK on proliferation of colorectal cancer cells. [file CAM4-14-e71027-s002.docx]
