# Supplementary material for: TWEAK/Fn14 Drives Tumor Progression and Is Associated With Poor Survival of Colorectal Liver Metastases With Replacement Growth Patterns
Source: Cancer Med. 2025 Jul 9;14(13):e71027. doi: 10.1002/cam4.71027 (PMC12238726; doi:10.1002/cam4.71027)
Supplement: Supplementary file 2 — Table S1. List of primary antibodies used in this study. [file CAM4-14-e71027-s001.docx]

**Table S1.** **List of primary antibodies used in this study**

| **Primary antibody** | **Sources** | **Dilution** |
| --- | --- | --- |
| Anti-vimentin antibody (sc6260) | Santa Cruz, CA, USA | IHC 1:100  WB 1:1000 |
| Anti-TWEAK antibody (PA5-96379) | Invitrogen, CA, USA | IHC 1:200 |
| Anti-Fn14 antibody (EPR3179) | Abcam, MA, USA | IHC 1:100  WB 1:1000 |
| Anti-CD8 antibody (M7103) | Dako, CA, USA | IHC 1:200 |
| Anti-IL-17A antibody (AF-317-NA) | R&D Systems, MN, USA | IHC 1:100 |
| Anti-CD163 antibody (NCL-L-CD163) | Leica Biosystems, IL, USA | IHC 1:200 |
| Anti-IκBα antibody (4812) | Cell Signaling Technology, MA, USA | WB 1:1000 |
| Anti-phospho-IκB (Ser32/36) antibody (9246) | Cell Signaling Technology, MA, USA | WB 1:1000 |
| Anti-NFκB p65 antibody (sc-8008) | Santa Cruz, CA, USA | WB 1:1000 |
| Anti-phospho-NFκB p65 (Ser536) antibody (3033) | Cell Signaling Technology, MA, USA | WB 1:1000 |
| Anti-Akt antibody (4691) | Cell Signaling Technology, MA, USA | WB 1:1000 |
| Anti-phospho-Akt (Ser473) antibody (9271) | Cell Signaling Technology, MA, USA | WB 1:1000 |
| Anti-mTOR antibody (2983) | Cell Signaling Technology, MA, USA | WB 1:1000 |
| Anti-phospho-mTOR(Ser2448) antibody (2971) | Cell Signaling Technology, MA, USA | WB 1:1000 |
| Anti-Snail antibody (ab216347) | Abcam, MA, USA | WB 1:1000 |
| Anti-Fibronectin/FN1 antibody (30903) | Cell Signaling Technology, MA, USA | WB 1:1000 |
| Anti-βactin antibody (5125) | Cell Signaling Technology, MA, USA | WB 1:2000 |
