# Supplementary material for: TWEAK/Fn14 Drives Tumor Progression and Is Associated With Poor Survival of Colorectal Liver Metastases With Replacement Growth Patterns
Source: Cancer Med. 2025 Jul 9;14(13):e71027. doi: 10.1002/cam4.71027 (PMC12238726; doi:10.1002/cam4.71027)
Supplement: Supplementary file 3 — Table S2. Univariate and multivariate analysis for disease free survival in patients with colorectal liver metastases. [file CAM4-14-e71027-s003.docx]

Table S2

Univariate and multivariate analysis for disease free survival in patients with colorectal liver metastases.

| Factors |  | Univariate analysis | | | Multivariate analysis | | |
| --- | --- | --- | --- | --- | --- | --- | --- |
|  |  | Hazard ratio | 95% CI | p-value | Hazard ratio | 95% CI | p-value |
| Age | < 60 | 1.48 | 0.96-2.28 | 0.08 |  |  |  |
|  | 60 ≤ |  |  |  |  |  |  |
| Sex | Female | 1.16 | 0.77-1.74 | 0.47 |  |  |  |
|  | Male |  |  |  |  |  |  |
| Timing of metastases | Synchronous | 1.65 | 1.09-2.48 | 0.02 | 1.69 | 1.05-2.72 | 0.03 |
|  | Metachronous |  |  |  |  |  |  |
| Primary lesion site | Rectum | 1.27 | 0.84-1.92 | 0.25 |  |  |  |
|  | Colon |  |  |  |  |  |  |
| Lymph node metastases of primary lesion | N (+) | 1.57 | 1.02-2.42 | 0.04 | 1.49 | 0.92-2.42 | 0.11 |
|  | N (-) |  |  |  |  |  |  |
| Tumor size (mm) | 50 mm ≤ | 1.19 | 0.75-1.88 | 0.46 | 1.19 | 0.72-1.94 | 0.50 |
|  | < 50 mm |  |  |  |  |  |  |
| Number of tumors | 5 ≤ | 1.70 | 1.08-2.68 | 0.02 | 1.23 | 0.73-2.08 | 0.43 |
|  | < 5 |  |  |  |  |  |  |
| Extrahepatic metastases | (+) | 1.42 | 0.86-2.34 | 0.16 | 1.40 | 0.80-2.47 | 0.24 |
|  | (-) |  |  |  |  |  |  |
| TWEAK/Fn14 | high/high | 1.84 | 1.19-2.85 | 0.01 | 1.76 | 1.11-2.77 | 0.02 |
|  | others |  |  |  |  |  |  |
| Adjuvant chemotherapy | (-) | 1.97 | 1.30-3.00 | <0.01 | 2.37 | 1.52-3.70 | <0.01 |
|  | (+) |  |  |  |  |  |  |
